# Supplementary material for: Increased Adipose Tissue Expression of Interferon Regulatory Factor (IRF)-5 in Obesity: Association with Metabolic Inflammation
Source: Cells. 2019 Nov 11;8(11):1418. doi: 10.3390/cells8111418 (PMC6912676; doi:10.3390/cells8111418)
Supplement: Supplementary file 1 [file cells-08-01418-s001.pdf]

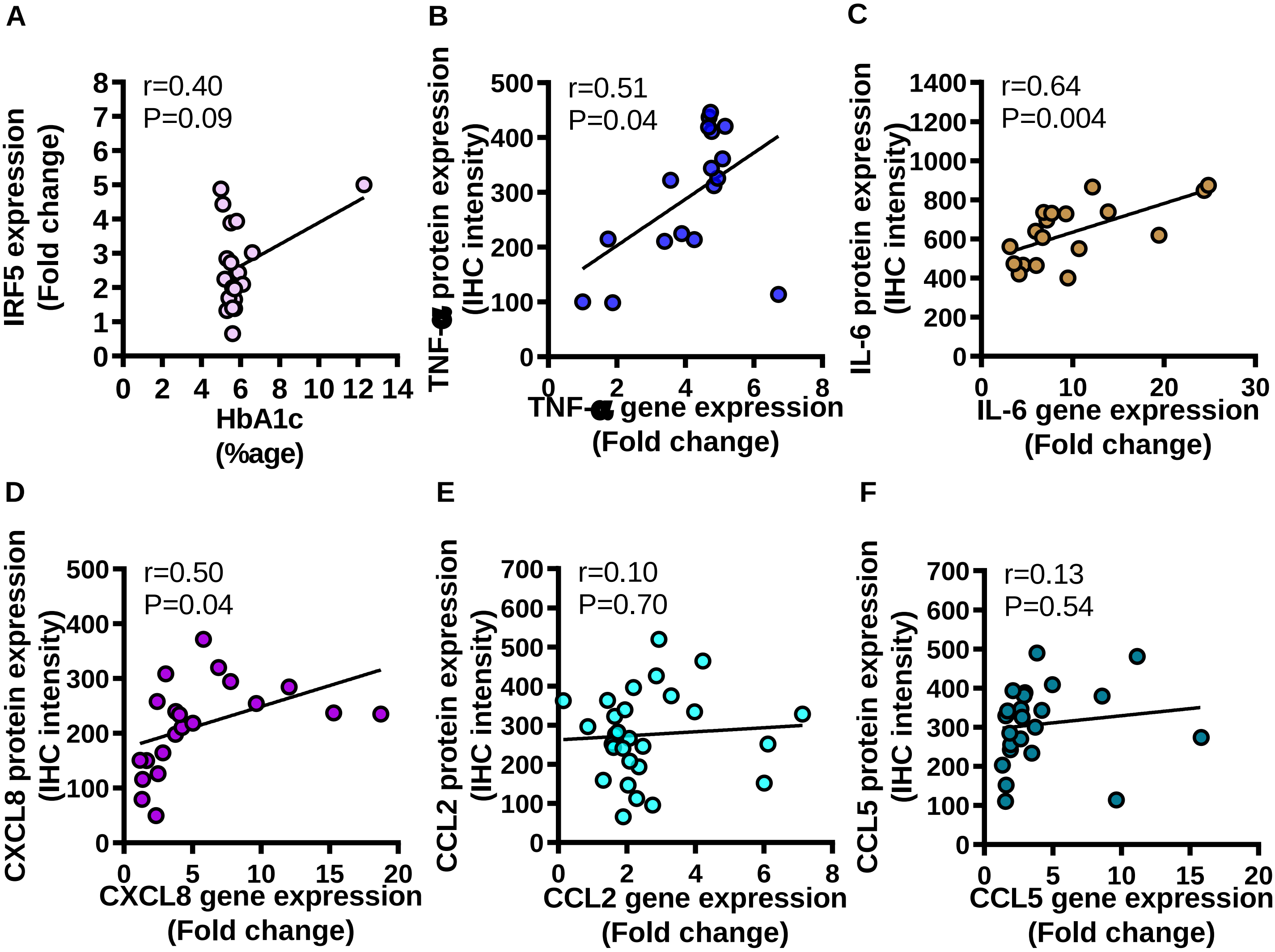

Figure S1. Adipose tissue gene expression (expressed as fold change) of IRF5, TNF-α, IL-6, CXCL8, CCL2, and CCL5 was determined by real-time RT-PCR. Adipose tissue protein expression (staining intensity expressed as arbitrary units) of TNF-α, IL-6, CXCL8, CCL2, and CCL5 was assessed by immunohistochemistry (IHC), as described in Materials and Methods. Plasma glycated hemoglobin (HbA1c) levels (expressed as %age) were measured by using Variant™ device (BioRad, Hercules, CA, USA). (A) Association between adipose IRF5 gene expression and HbA1c levels in 18 overweight non-diabetic individuals (BMI: 25-30 kg/m^2) is shown (r=0.40, P=0.09). (B) Correlation between adipose gene and protein expression of TNF-α in 17 individuals comprising 5 lean (BMI: <25 kg/m^2), 5 overweight (BMI: 25-30 kg/m^2) and 7 obese (BMI: >30 kg/m^2) is shown (r=0.51, P=0.04). (C) Correlation between adipose gene and protein expression of IL-6 in 18 individuals comprising 5 lean (BMI: <25 kg/m^2), 6 overweight (BMI: 25-30 kg/m^2) and 7 obese (BMI: >30 kg/m^2) is shown (r=0.64, P=0.004). (D) Correlation between adipose gene and protein expression of CXCL8 in 21 individuals comprising 6 lean (BMI: <25 kg/m^2), 8 overweight (BMI: 25-30 kg/m^2) and 7 obese (BMI: >30 kg/m^2) is shown (r=0.50, P=0.04). (E) Correlation between adipose gene and protein expression of CCL2 in 28 individuals comprising 6 lean (BMI: <25 kg/m^2), 12 overweight (BMI: 25-30 kg/m^2) and 10 obese (BMI: >30 kg/m^2) is shown (r=0.10, P=0.70). (F) Correlation between adipose gene and protein expression of CCL5 in 24 individuals comprising 6 lean (BMI: <25 kg/m^2), 8 overweight (BMI: 25-30 kg/m^2) and 10 obese (BMI: >30 kg/m^2) is shown (r=0.13, P=0.54).

Table S1. Primer IDs

Immune marker	Primer ID
IRF3	Hs01547283_m1
IRF5	Hs00158114_m1
TNF-α	Hs01113624_g1
IL-1β	Hs01555410_m1
IL-6	Hs00985639_m1
IL-18	Hs01038788_m1
IL-23A	Hs00900828_g1
CXCL8	Hs00174103_m1
CXCL9	Hs00171065_m1
CXCL10	Hs01124251_g1
CCL2	Hs00234140_m1
CCL5	Hs00982282_m1
CCL7	Hs00171147_m1
CCL11	Hs00237013_m1
CCL19	Hs00171149_m1
CCR1	Hs00928897_s1
CCR2	Hs00704702_s1
CCR5	Hs99999149_s1
TLR2	Hs01872448_s1
TLR4	Hs00152939_m1
TLR7	Hs01933259_s1
TLR8	Hs00152972_m1
TLR9	Hs00370913_s1
MyD88	Hs01573837_g1
IRAK-1	Hs01018347_m1
CD11c	Hs00174217_m1
CD68	Hs02836816_g1
CD86	Hs01567026_m1
CD163	Hs00174705_m1
CD302	Hs00994886_m1
GAPDH	Hs03929097_g1
